# Clinical comparison of conventional and additive manufactured stabilization splints

**DOI:** 10.1080/23337931.2018.1497491

**Published:** 2018-08-13

**Authors:** Christian Berntsen, Martin Kleven, Marianne Heian, Carl Hjortsjö

**Affiliations:** a Department of Prosthodontics, Faculty of Dentistry, University of Oslo, Oslo, Norway;; b Tannlab Dental Laboratory, Oslo, Norway

**Keywords:** Digital impression, hard occlusal stabilization splint, CAD-CAM

## Abstract

The aim of this study was to compare conventional and digital additive manufacturing of hard occlusal stabilization splints (SS) using technical and clinical parameters. 14 subjects were subjected to DC/TMD Axis I clinical examination protocol and Axis II questionnaire. The subjects underwent treatment with splints over a period of 12 weeks. All subjects underwent both conventional alginate impression and intraoral digital scanning. Seven subjects received conventional manufactured stabilization splints (CM-SS), and seven subjects received CAD-CAM additive manufactured stabilization splints (AM-SS). 12 subjects completed the 12 weeks follow-up period. The subjects significantly preferred digital intraoral scanning compared to conventional alginate impression. There was a significant difference in VAS between CM-SS and AM-SS. The mean VAS result was 15 for AM-SS and 42 for CM-SS, 0 represented excellent comfort and 100 very uncomfortable. This was significant. Splint manufacturing method had no influence on treatment outcome. There was no significant difference in mean delta change for unassisted jaw opening from baseline to 12 weeks between the two groups, for CM-SS it was 2 mm difference and for AM-SS the difference was 3 mm. All subjects in both treatment groups showed improved oral function. In this study, the scanning procedure is more accepted by the subjects than alginate impressions, however the first procedure was more time consuming.

## Introduction

Occlusal stabilization splints (SS) are often used as an interocclusal appliance for managing symptoms associated with temporomandibular disorders (TMD). TMD is a collective term for dysfunction and pain related to the masticatory musculature, the temporomandibular joints and associated structures [1]. These symptoms are often of low intensity and confined to within a specific area. Some patients on the other hand experiences chronic pain that last for at least 3–6 months [2] and are associated with behavioral, psychological and psychosocial factors [3]. Treatment with a hard interocclusal SS is scientifically recognized, have a low risk of side effects and is commonly used all over the world [4–6]. SS distributes the occlusal forces that occurs during teeth grinding or jaw clenching, by reducing the muscular tension and protecting teeth against wear. The therapeutic aim of SS is pain relief, and it is shown that a change in vertical dimension is one mechanism that can induce this. This mechanism is associated with changed functional patterns of the masticatory muscles and the position of the condyles [7].

The first SS were developed from a scandinavian precursor appliance by Ramfjord and Ash at the University of Michigan in the 1950s and 1960s. The term "Michigan splint" was first featured by Geering and Lang at the University of Bern, Switzerland, in an article published 1978 [8].

Hard SS have traditionally been manufactured of cold- or hot-processed polymethyl methacrylate (PMMA). Fabrication is accomplished on plaster casts based on an impression of the patients’ dental arches. The splints are thereafter tried intraorally and adjusted to obtain the desired adaptation to the teeth. This is a time consuming and expensive process for the dental technician, the dentist and the patient. Pore formation, large amount of residual monomer content and shrinkage during the production process are some key factors that can influence the structural quality of the SS [9].

Intraoral scanning is today an alternative to conventional impressions when doing prosthodontic treatment. Scanning has been shown to be time saving, and to increase treatment comfort. The precision of scanning procedure exceeds conventional impression techniques with the irreversible hydrocolloid material alginate [10,11]. Prosthodontic appliances are today often manufactured by computerized methods (CAD-CAM). Until today, fully digital manufacturing of acrylic splints has only been applied for splints used in guided implant treatment [12,13] and for orthodontic treatments [14]. A study by Salmi et al 2013 describes state of the art in digital process manufacturing of occlusal splints [15].

Early pioneering work have described the process of additive manufacturing to fabricate a mold for customized soft removable oral appliances [16]. This process has demonstrated adequate accuracy.

Epidemiological studies describe that TMD and orofacial pain occurs in all ages, but is most prevalent in patients aged between 20 and 50 years [17]. There is another epidemiological study among youths that found no difference in TMD-pain between male and female at the age of 12–13, but the difference between sexes increased with age [17]. The results from these studies corresponds with the demography of participants in our study.

Treatment of TMD with SS is a modality with well-established evidence. Treatment is reversible and the aim is to relax the muscles involved in the movement of the jaw, unload the TMJ and to protect the teeth against severe wear due to bruxism. Several systematic reviews and a number of RCTs have evaluated this treatment [6,18–20]. These studies suggest that SS will continue to be a part of TMD treatment in the foreseeable future. The new digital CAD-CAM manufacturing processes are of great interest for the dental profession, increasing treatment possibilities giving the TMD patients an optimal, comfortable and possible more economical treatment.

The aim of this randomized controlled trial was to compare conventional production and CAD-CAM additive production of hard occlusal SS using technical and clinical parameters. The null-hypothesis was that there were no differences between traditionally manufactured splints using alginate impressions and PMMA and digital manufactured splints using intraoral scanning and an additive technique when it comes to the subjects’ experience, outcome of the treatment, time used in the dental chair for impression taking and adjustment of the SS and finally comparing the internal fit of the conventional and digital manufactured splints.

## Materials and methods

### Study design

The study was approved by the Regional Committee for Medical and Health Research Ethics, South East Norway (REK sør-øst 2016/1548 B).

The study was a double blinded randomized controlled trial (RCT). The blinding was achieved by taking both digital and conventional impressions of all participants. Manufacturing method was randomly decided at the dental laboratory known only to the dental technician manufacturing the splint. The scientifically accepted diagnostic criteria for temporomandibular disorders (DC/TMD) was used [20] in order to identify subjects in need for treatment with SS. All subjects were treated by the same dentist (CB) under the supervision of a senior specialist in prosthodontics (CH).

### Registrations

Time used for conventional and digital impressions. Time used for necessary individual adjustments of the splints as well as, occlusion- and articulation contacts. The time recorded in minutes used. The subjects own experience regarding the different impression techniques and overall experience of the SS-treatment registered using a visual analog scale (VAS).

The fit of the splints on the occlusal surfaces on teeth 16 and 26 was recorded. This was done by the use of a replica technique described by Boening K et al. [21]. The technique has been evaluated by Laurent et al. [22] to give a good measurement on the luting thickness between a crown and the tooth. The inner part of the splints in the regions 16 and 26 were filled with a-silicon (Provil Novo, medium fast set, Heraeus). The splint was then placed in position with a firm pressure until the silicon had set. This silicon had a yellow color. The splint was carefully removed from the teeth with the silicon fixed inside the splint. This thin layer of silicon, which represent the space of the inner part of the splint and the teeth, was then stabilized with another a-silicon (Provil Novo putty, fast set, Heraeus). This had a blue color. The silicon was then removed in one piece from the splint, and this represented then a complete shape of the teeth 16 and 26 together with the space in between represented by the yellow a-silicon. These specimens were then sliced into one mm thick pieces in bucco-palatinal direction with a sharp racer blade. The slices were then observed in light emission microscope (Leica DMBRE, Germany) with 2,5x. Each slice was photographed in three places through the microscope. These pictures were then examined using a software (Image J version 1,51r). The thickness of the yellow a-silicon was then measured on three places, the most prominent buccal and palatinal cusps and the deepest fossa, in total six measurements on each subject and in total on all 14 subjects 84 measurements were done.

### Inclusion and exclusion criteria

Inclusion criteria: TMD diagnosis myalgia.

Exclusion criteria: Severe jaw functional limitations, severe somatic symptoms, depression, anxiety, removable dentures, trauma of recent date towards face, head or neck, dentoalveolar pathology or ongoing treatment related to TMD.

### Subjects

Subjects were selected from referrals to The Department of Prosthetic Dentistry and Oral Function at The Faculty of Odontology, University of Oslo, and by e-mail advertisement directed to the staff and students at the faculty of Odontology. 14 subjects met the inclusion criteria and were offered to participate in the study. 13 females and 1 male. The average age was 40,5 years (21–55). They were all given written information about the study, and an informed consent was obtained prior to commencing the study. As part of the written information, the subjects were informed that they could withdraw from the study at any time without having to explain the reason. The subjects were randomly assigned into two groups at the dental laboratory by the dental technicians. Group one received conventional SS (CM-SS) and group two received additive manufactured SS (AM-SS).

#### Diagnostic criteria for temporomandibular disorders (DC/TMD)

DC/TMD is an evidence-based diagnostic procedure of orofacial pain and TMD [23–25].

The clinical examination protocol according to Axis I, and the questionnaire form following the protocol of Axis II was used. Pain free jaw opening and maximum unassisted jaw opening were recorded before during and after treatment. If pain was present before opening, the subject was asked to open as wide as possible without increasing the level of pain. Jaw opening and maximum unassisted opening were recorded by measuring the inter-incisal distance between a maxillary and mandibular anterior reference tooth in mm. Two instruments from the Axis II questionnaire form were used to compare treatment outcome. Jaw Functional Limitation Scale - 20-item - JFLS [26] and Graded Chronic Pain Scale Version 2.0 - GCPS [26].

The mean delta values for jaw opening, JFLS and GCPS were calculated by subtracting the baseline value from the 12 weeks value.

### Subjects self-reported comfort

The VAS was a 0–100 mm straight line. 0 represented excellent comfort and 100 very uncomfortable. A visual analog scale (VAS) was used to report the subjective experience of alginate impression, digital scanning of the dental arches and after adjustment of the SS. A final VAS registration was performed after treatment completion to assess the overall treatment experience.

### Clinical procedure

Over a period of four months the subjects were asked to meet five times at the dental clinic.

#### First visit:

The subjects were interviewed and examined according to DC/TMD protocol, and a panoramic x-ray taken at the department of radiology.

All subjects were given information about their condition and instructions in jaw stretching exercises. Information about the potential effect of using a SS was given and the subjects consented to treatment with SS.

The subjects were first subjected to digital intraoral scanning followed by alginate impression. The digital recordings of the mandibular and maxillary arches were acquired using a TRIOS intraoral scanner (3Shape A/S, Copenhagen, Denmark). Intercuspidal occlusion was recorded. The scanning time from start until approved scan was recorded using a digital timer. Before the alginate impression the VAS registration of the scanning experience was recorded.

Conventional alginate impressions (Alginoplast fast set, Heraeus Kulzer, Hanau, Germany) of the upper and lower dental arches using perforated stainless-steel trays (Ultradent/Topdent) and alginate mixer (AM 501, Hauschild & Co KG, Hamm, Germany). Bite index (Alminax Rite Bite Index, Kemdent, Swindon, England) was taken in retruded position.

The time of the conventional impression was recorded from the start of mixing water into the alginate powder until the bite registration was completed. Immediately after the conventional impression procedures, the subjects marked their subjective experience on a VAS schedule.

The digital files were transmitted and the impressions were send to the collaborating dental laboratory.

#### Second visit:

The SS were controlled for retention, contacts in occlusion and articulation, adjustment done when necessary and then delivered to the subjects. Blue occlusion foil (Blue Radar, 65 µ, Nordin, Montreux, Switzerland) was used to mark mandibular teeth in contact with the occlusal plane of the splint. The try-in time and time of occlusal adjustment were recorded using the digital timer.

The subjects’ SS adjustment experiences were recorded on a VAS schedule.

The subjects were instructed in use of the splint. The splint was to be used every night for approximately twelve weeks, but no more than twelve hours per day.

#### Third visit:

Functional and adaption control of the SS. The splints were controlled after two weeks of function, if necessary further minor adjustments were made to the SS.

#### Fourth visit:

After a further 6 weeks, the subjects underwent a clinical DC/TMD examination and the use of SS were registered.

#### Fifth visit:

After three months, the subjects had a final control. A third DC/TMD examination was performed. The subjects scored their overall SS treatment experience on a VAS schedule. The subjects were informed about the possibility of continuing using their SS provided follow up by their dentist at their annual recall appointments.

### Splint manufacturing

The CM-SS was made from heat polymerized PMMA with press technique (Vertex Rapid Simplified, Vertex-Dental, Soesterberg, The Netherlands).

The AM-SS were manufactured by the use of computer aided design – computer aided manufacturing (CAD-CAM). For the CAD process an appliance designer software was used (3Shape Dental Designer 2016, Splint program, 3Shape A/S, Copenhagen, Denmark). For the CAM process methacrylate based resin (IMPRIMO LC Splint light-curing resin at the base) was used. This material is specifically engineered for fabrication of high-precision dental occlusal splints and drilling templates. The additive manufacturing machine used for vat photopolymerization was 3 D printer IMPRIMO 90 LC Splint, Scheu Dental GmbH, Iserlohn, Germany. The source used was high performance UV LED radiation source (382 nm) with DLP technology using 385 nm. The scaling factor in the process was 1:1. The supports were manual removed by hand after the completed curing and cleaning with IPA. For finishing the splints were first prewashed 10 min with IPA, then a second wash for 15 min before UV curing for additional 15 min. Vat photopolymerization is a process where laser hardens curable photopolymer resin layer by layer. Each layer is cured before the next layer is applied and cured. This process is repeated until the splint is completely build.

The CM-SS and AM-SS were designed in accordance to the following guidelines: All maxillary teeth covered, flat and smooth occlusal surfaces, balanced and similar bilateral contacts with the mandibular teeth and cuspid guidance during lateral movements and even bilateral incisal contacts during protrusion of the mandible.

### Statistical analysis

Kolmogorov-Smirnov normality test failed a non-parametric method. Wilcoxon Signed Rank test was used to compare time and VAS score for alginate- and digital impression and jaw opening. Mann-Whitney U test was used to used compare adjustment time, VAS experience, assisted and un-assisted jaw opening between the two groups. The measurements of the internal fit of the splints were analyzed with an independent T-test, The data was analyzed with SPSS version 25 (SPSS Inc. Chicago, Illinois, USA). The mean differences were considered significant at the 0.05 level.

## Results

**Figure 1.**
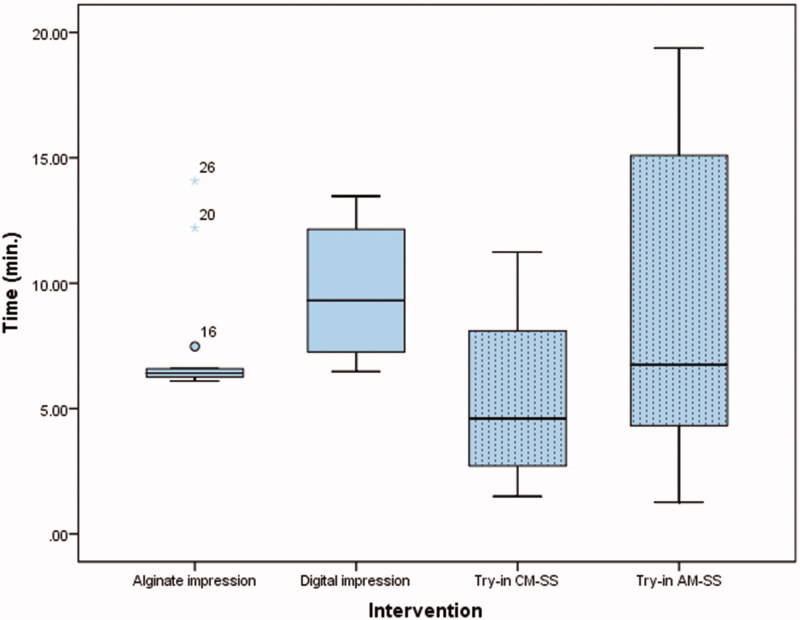
Box-plot showing the median time recorded for alginate impressions and bite registration, scanning procedure and try-in extradition of the splints.

**Figure 2.**
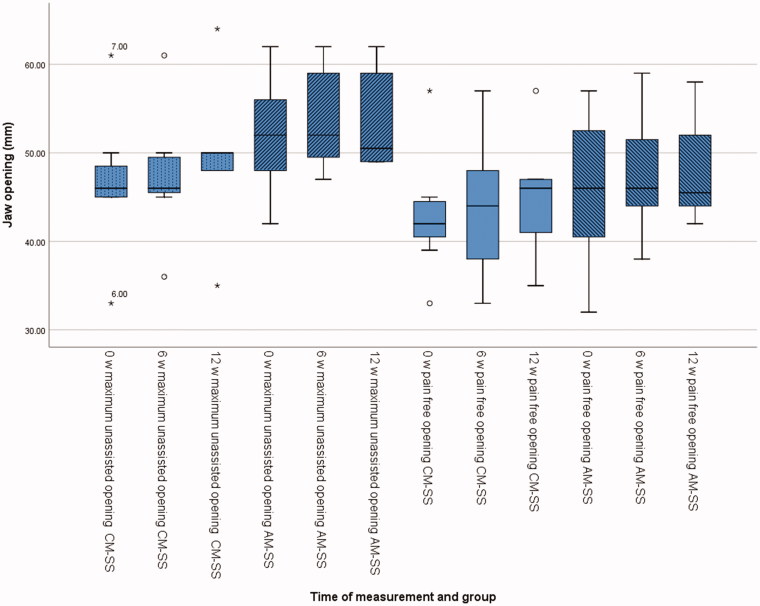
Box-plot showing the maximum unassisted and pain-free jaw opening at baseline, 6 weeks and at 12 weeks for both splint groups.

**Figure 3.**
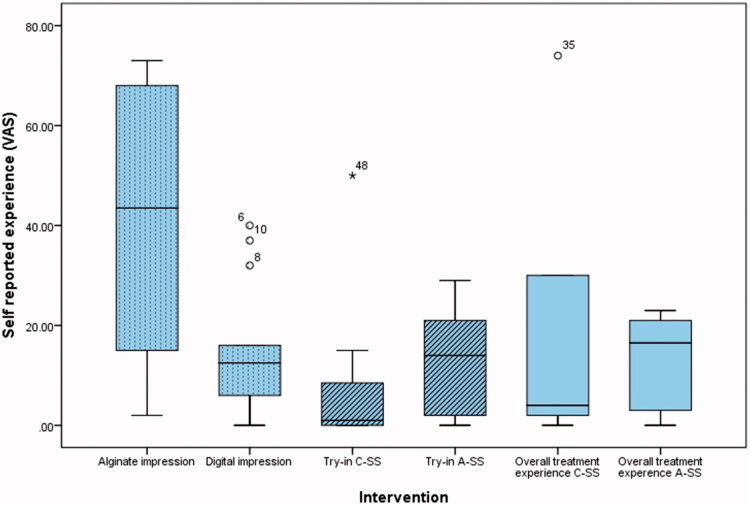
Box-plot showing the VAS results for all the recorded procedures, alginate impression, digital impression, try-in and overall treatment experience for both splint groups.

Time recorded for alginate impressions and bite registration, scanning procedure and try-in of the splints are presented in Figure 1. The maximum unassisted and pain-free jaw opening at baseline, 6 weeks and at 12 weeks are presented in Figure 2. The VAS results for all procedures and the use of SSs are presented in Figure 3. The main results are presented in Table 1. There were two significant differences between CM-SS and AM-SS. First, when comparing the impressions technique in favor of AM-SS, and second when comparing time needed for impressions in favor of CM-SS.

**Table 1.** Presenting the difference between CM-SS and AM-SS groups. Delta pain free and maximum jaw opening presented in mm. VAS impression, Try-in and overall use score represents the score on a straight line between 0–100 mm were 0 represented excellent comfort and 100 very uncomfortable. The time impression and try-in presented in minutes and Delta JFLS and GCPS changes in score.

	CM-SS			AM-SS				
	n	Mean	SD	n	Mean	SD	P-value	Statistical Method
Delta pain free opening 6w	6	1	2	7	3	4	ns	Student t-test
Delta pain free opening 12w	6	2	2	6	3	6	ns	Student t-test
Delta maximum unassisted opening 6w	6	1	2	7	3	4	ns	Mann-Whitney U Test
Delta maximum unassisted opening 12w	6	3	1	6	3	4	ns	Student t-test
VAS impression	14	42	25	14	15	12	0.004	Wilcoxon Signed Rank Test
VAS Try-in	7	10	19	7	13	12	ns	Mann-Whitney U Test
VAS overall use	6	19	29	6	13	10	ns	Mann-Whitney U Test
Time impression	14	7	2	14	10	2	0.004	Wilcoxon Signed Rank Test
Time Try-in	7	6	4	7	9	7	ns	Student t-test
Delta JFLS-20	6	−12	18	6	−8	14	ns	Student t-test
Delta GCPS	6	−2	9	6	−1	8	0.000	Student t-test

The average thickness of the gap between the teeth 16 and 26 and the internal surface of the splints were 603 µm for CM-SS and 570 µm for AM-SS. The difference was not significant.

One subject from each group did not complete the treatment over 3 months. The first subject was withdrawn from the study after 6 weeks following recommendations by the responsible clinician due to increased myofacial pain. The second subject did not attend the final control. This subject was interviewed by telephone and had not been using the splint since the 6^th^ week control due to reduced symptoms.

The remaining 12 subjects all completed the follow-up period.

## Discussion

The null hypothesis was partially proven wrong. The essential findings of this study are that the subjects self-reported experience for intra oral scanning was significant better when compared to conventional impressions, meaning that intraoral scanning was perceived to be more comfortable compared to alginate impressions. Another significant finding was the different times needed for digital and conventional impressions. There were no significant differences in treatment outcome. A study by Gjelvold et al. [27] comparing intraoral digital impression technique with conventional impression technique showed similar results. They treated forty-two subjects with tooth-supported fixed dentures and evaluated and compared the procedure time, dentists’ and patients’ assessments using a visual analog scale(VAS) between the two groups. They reported the mean impression times for digital and conventional impressions technique were 7.33 ± 3.37 and 11.33 ± 1.56 respectively. The mean VAS scores for patients’ assessment of discomfort were 6.50 ± 5.87 and 44.86 ± 27.13 respectively (the value 100 meaning the most discomfort). They also concluded that the digital technique was more efficient and convenient than the conventional impression technique.

This study wanted to analyze the potential benefits of digital manufacturing of stabilization appliances. The first thing was to compare the time spent for conventional alginate impressions and digital recordings with an intraoral scanner. The difference between the two groups are significant, and our study suggests that conventional technique with alginate impressions is less time consuming. One factor that can explain the difference is the operators scanning skills compared to the experience with taking alginate impressions. The operator had been instructed and received practical learning in advance of the study, but had not been using intraoral scanner regularly over a longer period of time.

The scanning procedure is a manageable and predictable procedure, and has potential many advantages. This is also shown in the results of the study, that even though the clinician was unexperienced with intraoral scanning, the subjects preferred scanning instead of conventional impression technique. This demonstrates clearly one of the main advantages of modern digital dental techniques.

A notable drawback using intraoral scanning was the possibility to scan to difficult positioned teeth. Two subjects were considerably more time consuming to scan than others. These subjects were the same that had difficulties with impressions. This due to partial erupted and malposition of 3^rd^ molars. This may be explained by mainly to reasons the size of the intra oral scanning head making the positioning difficult.

The average time for try-in of the splints was within a minute for both groups, and there was no significant difference. Average time spend on occlusal individual adjustment was slightly shorter for group 1 with CM-SS than group 2 with AM-SS. There was no significant difference between the groups.

The subjects were examined according to the DC/TMD protocol three times during the study. At the first visit, after about six weeks using the appliance and at the final control after about 12 weeks. All had been diagnosed with myalgia at the first visit, given information about their condition and advised to perform jaw stretching exercises according to guidelines in the management of orofacial pain and recommended treatment with SS [28]. Myalgia was chosen as an inclusion criteria since it is the most common TMD diagnose that occurs in 80% of cases [28,29]. Myalgia is defined as pain that is recognizable in the jaw muscles and is affected by jaw movement, function or parafunction. Valid sensitivity and specificity have been acknowledged for myalgia as a diagnosis.

Jaw opening is one clinical sign that is easily measured and can describe the improvement of TMD. One diagnostic test for myalgia is that the pain can be reproduced through provocation. Opening of the jaws is such a provocation test together with palpation of m. temporalis and m. masseter. [23,30]. The average jaw opening at the first clinical examination was 45 mm and maximal jaw opening was 49 mm. After about six weeks the same figures were 46 mm and 51 mm, and after about twelve weeks 47 mm and 51 mm. One subject had small jaw opening during the whole treatment and did not respond with increased jaw opening, but even so experienced less TMD symptoms. Because most of the participants from the start had a normal range of jaw opening, it can be concluded that all patients that managed to use the splints regularly over three months, experienced less TMD symptoms. The small changes in TMD symptoms were confirmed by the changes in JFLS and GCPS scores.

After the end of the treatment period, the subjects were asked to report their subjective experience of the treatment of their TMD. The average value was 16 on a 100 mm long VAS. This is a good marker that the treatment of TMD, is well appreciated among people with this condition, and it is important to offer treatment to patients with TMD.

The two study groups AM-SS and CM-SS represent an all-digital work flow and an all conventional work flow. The scope of this study was however the subjects experience and the clinical aspects of the treatment leaving out dental laboratory aspects. A limitation within this study is all the aspects of the work flows at the dental laboratory i.e. not fully comparing the two manufacturing techniques with each other. However, the dental technicians reported that in this study the CM-SS production took approximately 3 h and for AM-SS production took 2 h respectively. Another benefit reported is that the first digital impression may be saved and additional splints may be manufactured without a new impression. If wanted the design can be altered by only small adjustments in the CAD program. The cost for material is much lower for pressed PMMA SS than for AM-SS. There are additional costs for software and software licenses, 3 D printer and the resins used. As for comparable industrial processes, volume lowers the cost.

In this study, we have measured the precision of the splints by looking at the internal fit of the splints on teeth 16 and 26. The results show that there is no difference in the internal fit between the two groups. This means that the retention of AM-SS is comparable to CM-SS. There were limitations that can have affected the results, with this method of measuring. The cutting of the specimens, air bubbles between the two impression materials in the repliqua and relatively few points of measurements. Another study by Salmi et al. [31] have investigated the accuracy of producing medical models between different materials, and found that the accuracy of PolyJet was higher than for selective laser sintering or three-dimensional printing.

Both CM-SS and AM-SS reported good comfort after three months use. This together with the DC/TMD scores and improved oral functions indicates equivalent treatment effects for both splint types.

Recently Pho Duc et al. [32]) published a similar study, but with longer follow-up and larger sample size with 32 subjects completing the study. This study found no differences in splint adjustment time or in patient's preference in favor of one technique over another. CAD/CAM splint versus conventional stabilization splint were equally efficacious with no difference in between them. This regarding pain and TMJ functional assessments. A fundamental difference between this study and our study is that we compare an all-digital workflow starting with intraoral scanning and compare it with a conventional workflow, while Pho Duc et al. only present a partial digital workflow using the CAD/CAM technique on stone casts and compared with conventional workflow. This might explain why the subjects in Pho Duc et al.'s study did not prefer one technique over another and why our subjects reported otherwise.

There were several limitations with this study. The number of participants in the study was low, and the balance between female and male were not ran optimal range for extensive TMD studies. The average age in the group were also higher than in larger TMD studies. The results of the study showed only difference of the time to perform and patient's comfort. If larger number of participants had been included in the study design from the beginning, the results of the different elements of the study could have been different.

## Conclusion

Within the limitations of this study it can be concluded that intraoral digital scanning was the significantly preferred impression method among the subjects. Full arch intra oral scanning took longer time compered to alginate impression. In regard to the treatment of TMD patient with mild myalgi, no methods were superior to the other. Further studies with larger groups of participant and improved study design are needed to confirm these results.

## References

[1] Bell WE Clinical management of temporomandibular disorders. Chicago: Year Book Medical Publishers; 1982 p. 128–171.

[2] Parker MW, Holmes EK, Terezhalmy GT Personality characteristics of patients with temporomandibular disorders: diagnostic and therapeutic implications. J Orofacial Pain. 1993;7:337–344. 8118435

[3] Mersky H Classification of chronic pain descriptions of chronic pain syndromes and definitions of pain terms. Pain. 1986;27:1–225. 3461421

[4] Klasser GD, Greene CS Oral appliances in the management of temporomandibular disorders. Oral Surg Oral Med Oral Pathol Oral Radiol Endod. 2009;107:212–223. 19138639 10.1016/j.tripleo.2008.10.007

[5] List T, Axelsson S Management of TMD: evidence from systematic reviews and meta-analyses. J Oral Rehabil. 2010;37:430–451. 20438615 10.1111/j.1365-2842.2010.02089.x

[6] Friction J, Look JO, Wright E, et al. Systematic review and meta-analysis of randomized controlled trials evaluating intraoral orthopaedic appliances for temporomandibular disorders. J Orofac Pain. 2010;24:237–254. 20664825

[7] Schindler HJ, Hugger A, Kordaz B, et al. Splint therapy for temporomandibular disorders: basic principles. J CranioMand Func. 2014;6:207–230.

[8] Geering AH, Lang NP Die Michigan-Schiene, ein diagnostiches und therapeutisches Hilfsmittel bei Funktionsstorungen im kaufsystem. Herstellung im Artikulator und Eingliederung am Patienten [The Michigan splint, a diagnostic and therapeutic aid when functional symptoms in the jaws]. Schweiz Monatsschr Zahnheilkd. 1978;88:32–38. 272057

[9] Dedem P, Türp JC Digital Michigan splint: from intraoral scanning to plasterless manufacturing. Int J Comput Dent. 2016;19:63–76. 27027103

[10] Zimmermann M, Koller C, Rumetsch M, et al Precision of guided scanning procedures for full-arch digital impressions *in vivo*. J Orofac Orthop. 2017;78:466–471. 28733810 10.1007/s00056-017-0103-3

[11] Ender A, Mehl A *In-vitro* evaluation of the accuracy of conventional and digital methods of obtaining full-arch dental impressions. Quintessence Int. 2015;46:9–17. 25019118 10.3290/j.qi.a32244

[12] Reiz SD, Neugebauer J, Karapetian VE, et al. Cerec meets Galileos: integrated implantology for completely virtual implant planning. Int J Comput Dent. 2014;17:145–157. 25098162

[13] Ritter L, Palmer J, Bindl A, et al. Accuracy of chairside-milled CAD/CAM drill guides for dental implants. Int J Comput Dent. 2014;17:115–124. 25098159

[14] Garino F, Garino GB, Castroflorio T The iTero intraoral scanner in Invisalign treatment: a two-year report. J Clin Orthod. 2014;48:98–106. Feb 24763683

[15] Salmi M, Paloheimo KS, Tuomi J, et al. A digital process for additive manufacturing of occlusal splints: a clinical pilot study. J R Soc Interface. 2013;10:20130203. 23614943 10.1098/rsif.2013.0203 PMC3673156

[16] Salmi M, Tuomi J, Sirkkanen R, et al. Rapid tooling method for soft customized removable oral appliances. Open Dent J. 2012;6:85–89. 22615719 10.2174/1874210601206010085 PMC3355367

[17] Drangsholt M Temporomandibular pain In: Crombie I, Croft P, Linton S, et al., editors. Epidemiology of pain. Seattle: IASP Press; 1999 p. 203–234.

[18] Nilsson IM, List T, Drangsholt M Prevalence of temporomandibular pain and subsequent dental treatment in Swedish adolescents. J Orofac Pain. 2005;19:144–150. 15895837

[19] SBU. Metoder for behandling av långvarig smärta. Rapport nr 2006; 177.

[20] Al-Ani Z, Gray RJ, Davies SJ, et al. Stabilization splint therapy for the treatment of temporomandibular myofascial pain: a systematic review. J Dent Edu. 2005; 69:1242–1250. 16275687

[21] Boening K, Wolf B, Schmidt A, et al. Clinical fit of procera AllCeram crowns. J Prosthet Dent. 2000;84:419–124. 11044849 10.1067/mpr.2000.109125

[22] Laurent M, Scheer P, Dejou J, et al Clinical evaluation of the marginal fit of cast crowns-validation of the silicone replica method. J Oral Rehabil. 2008;35:116–122. 18197844 10.1111/j.1365-2842.2003.01203.x

[23] Schiffman EL, Truelove EL, Ohrbach R, et al. The research diagnostic criteria for temporomandibular disorders. I: overview and methodology for assessment of validity. J Orofac Pain. 2010;24:7–24. 20213028 PMC3157055

[24] Ohrbach R, Gonzalez Y, List T, et al. Diagnostic criteria for temporomandibular disorders (DC/TMD) clinical examination protocol: Version June 2, 2013 Available from: www.rdc-tmdinternational.org

[25] Schiffman E, Ohrbach R, Truelove E, et al Diagnostic criteria for temporomandibular disorders (DC/TMD) for clinical and research applications: recommendations of the international RDC/TMD consortium network* and orofacial pain special interest group†. J Oral Facial Pain Headache. 2014;28:6–27. 24482784 10.11607/jop.1151 PMC4478082

[26] Ohrbach R, Larsson P, List T The jaw functional limitation scale: development, reliability, and validity of 8-item and 20-item versions. J Orofac Pain. 2008;22:219–230. 18780535

[27] Gjelvold B, Chrcanovic BR, Korduner EK, et al. Intraoral digital impression technique compared to conventional impression technique. A randomized clinical trial. J Prosthodont. 2016;25:282–287. 26618259 10.1111/jopr.12410

[28] Wänman A, Ernberg M, List T Guidelines in the management of orofacial pain/TMD. An evidence-based approach. Nor Tannlegeforen Tid. 2016;126:104–112.

[29] Peck CC, Goulet J-P, Lobbezoo F, et al. Expanding the taxonomy of the diagnostic criteria for temporomandibular disorders (DC/TMD). J Oral Rehabil. 2014 ;41:2–23. 24443898 10.1111/joor.12132 PMC4520529

[30] List T, Dworkin SF Comparing TMD diagnosis and clinical findings at Swedish and US TMD centers using research diagnostic criteria for temporomandibular disorders. J Orofac Pain. 1996;10:240–253. 9161229

[31] Salmi M, Paloheimo K-S, Tuomi J, et al. Accuracy of medical models made by additive manufacturing (rapid manufacturing). J Craniomaxillofac Surg. 2013;41:603–609. 23333490 10.1016/j.jcms.2012.11.041

[32] Pho Duc JM, Hüning SV, Grossi ML Parallel randomized controlled clinical trial in patients with temporomandibular disorders treated with a CAD/CAM versus a conventional stabilization splint. Int J Prosthodont. 2016;29:340–350. 27479339 10.11607/ijp.4711

[33] Von Korff M Assessment of chronic pain in epidemiologiacal and health services research: empirical bases and new directions In: Turk DC, Melzack R, editors. Handbook of pain assessment. 3rd ed New York: Guilford Press; 2011 pp. 455–473.

